# Supplementary figures and images for: NuA4 and SAGA acetyltransferase complexes cooperate for repair of DNA breaks by homologous recombination
Source: PLoS Genet. 2021 Jul 6;17(7):e1009459. doi: 10.1371/journal.pgen.1009459 (PMC8284799; doi:10.1371/journal.pgen.1009459)

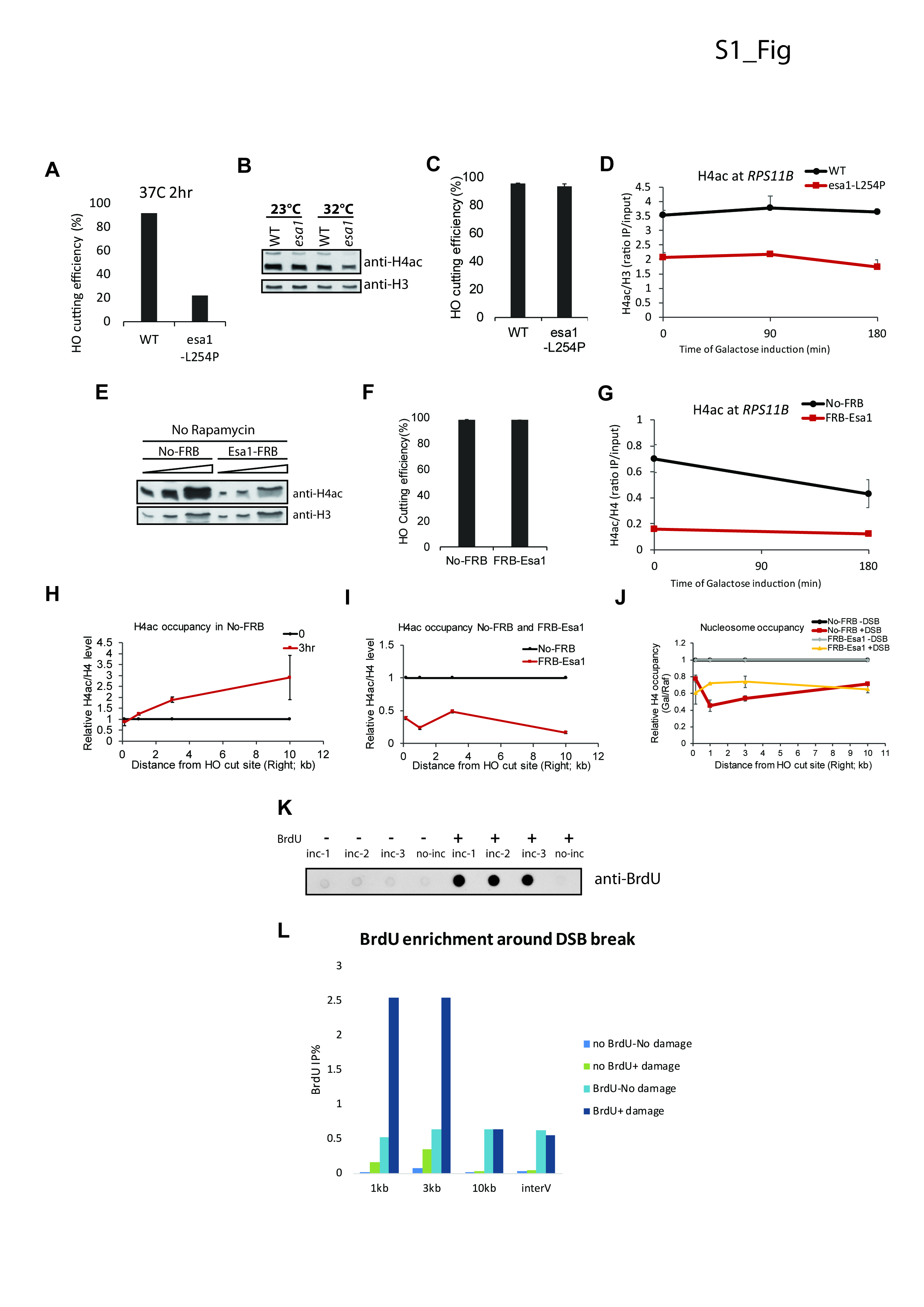

Supplement: S1 Fig — Related to Fig 1. A) Percentage of HO cutting efficiency in WT and esa1-L254P mutant after 2hr 37°C treatment followed by 3hr galactose induction at 37°C. B) Western blot (WB) of whole cell extract (WCE) from WT and esa1-L254P cells treated with 23°C or 32°C for 3hr. Antibodies used are penta-H4 acetylation (Upstate, 06–946) and H4 (Abcam, Ab7311). C) Percentage of HO cutting efficiency in WT and esa1-L254P cells after 3hr galactose induction at 32°C (based on qPCR across the cut site normalised to control locus). D) ChIP-qPCR of H4ac signal at the RPS11B control locus in WT and esa1 ts strains during galactose induction of HO nuclease, showing no increase (ratio of H4ac/total H3%IP/input, error bars are range of biological replicates). E) Western blot (WB) of H4 acetylation and H3 with whole cell extract (WCE) from indicated strains grown in YPD without rapamycin treatment, showing the non-specific effect of Esa1 tagging at its C-terminus. F) HO cutting efficiency as above in No-FRB and FRB-Esa1 cells after 30min of galactose induction in presence of rapamycin (depleted conditions, no change is seen after 3hrs either). G) ChIP-qPCR of H4ac signal as above at the RPS11B control locus in No-FRB and FRB-Esa1 strains during galactose induction of HO nuclease. H) Relative H4ac/total H4 level by ChIP-qPCR (ratio of % IP/input) in No-FRB cells after 3hr galactose induction in presence of rapamycin. Values at the different locations near the DSB at time 0 was normalized to 1, showing the increase of H4 acetylation upon HO cut. I) Relative H4ac/total H4 level by ChIP-qPCR (ratio of % IP/input) in No-FRB and FRB-Esa1 cells after 3hr galactose induction in presence of rapamycin. Values at the different locations near the DSB in No-FRB cells were normalized to 1, showing the loss of H4 acetylation induction in FRB-Esa1 cells. J) Relative total H4 level (nucleosome occupancy) by ChIP-qPCR in No-FRB and FRB-Esa1 cells after 0 and 3hr galactose induction in presence of rapamycin. [file pgen.1009459.s001.tif]

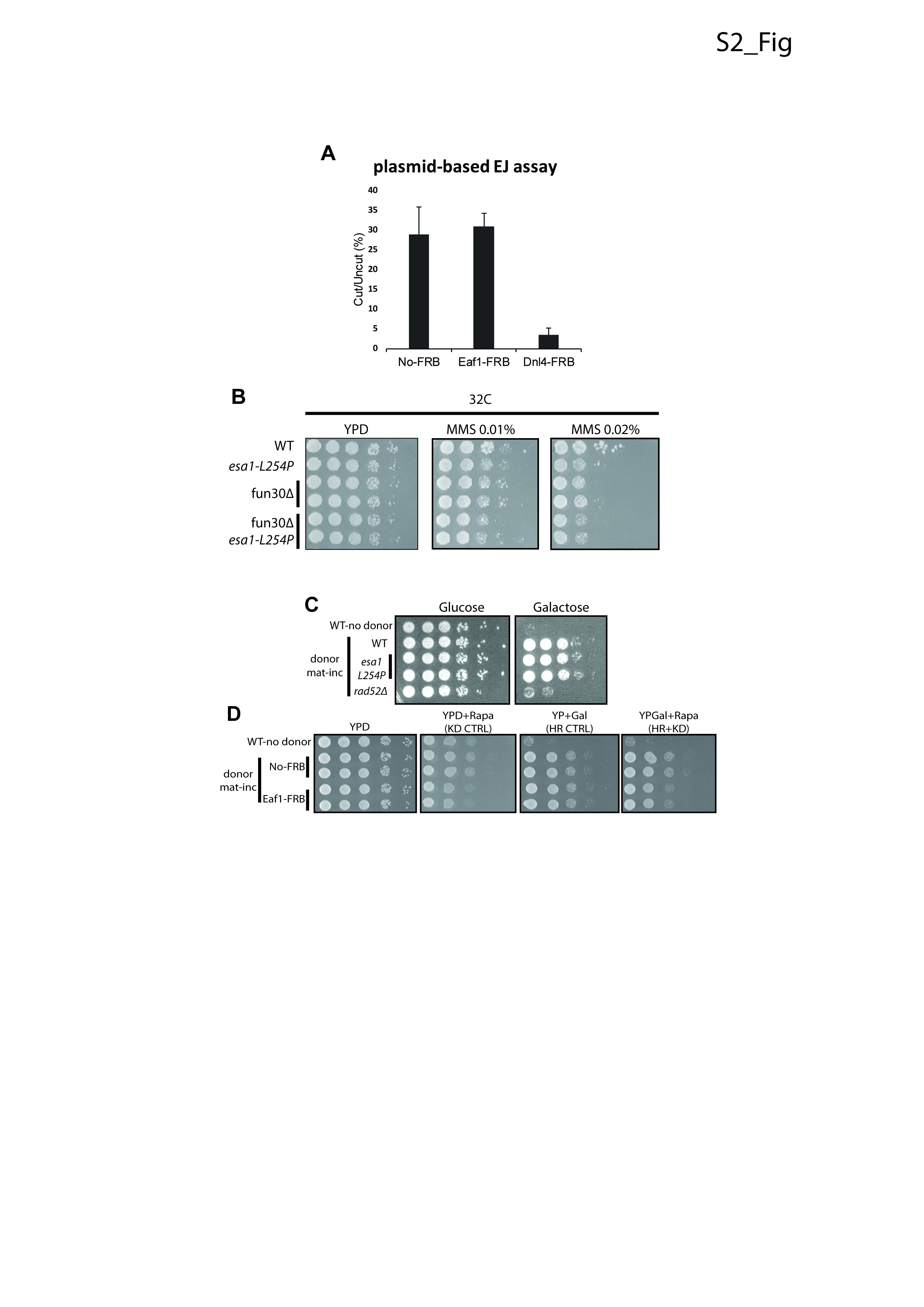

Supplement: S2 Fig — Related to Figs 2 and 3. A) Plasmid-based NHEJ assay using indicated strains transformed with cut or uncut plasmids, plated on 1μg/ml rapamycin-containing SCSM-URA solid medium and grown at 30°C. Error bars represent standard errors from biological triplicates. B) 10-fold serial dilutions of indicated strains spotted on solid medium without or with indicated amounts of MMS and grown at 32°C, showing synthetic sickness/interaction between esa1 ts mutant and fun30 deletion. C-D) 10-fold serial dilutions of indicated strains were spotted on YPD or YP containing 2% galactose to induce HO break and recombination with the mat-inc donor locus (measured by survival, see system schematic in Fig 5A). The esa1 ts mutant shows no defect in HR at 32°C (rad52 deletion is used as positive control) (C). The Eaf1-FRB strain does not show HR defect either in presence of 1μg/ml rapamycin grown at 30°C (D). (TIF) [file pgen.1009459.s002.tif]

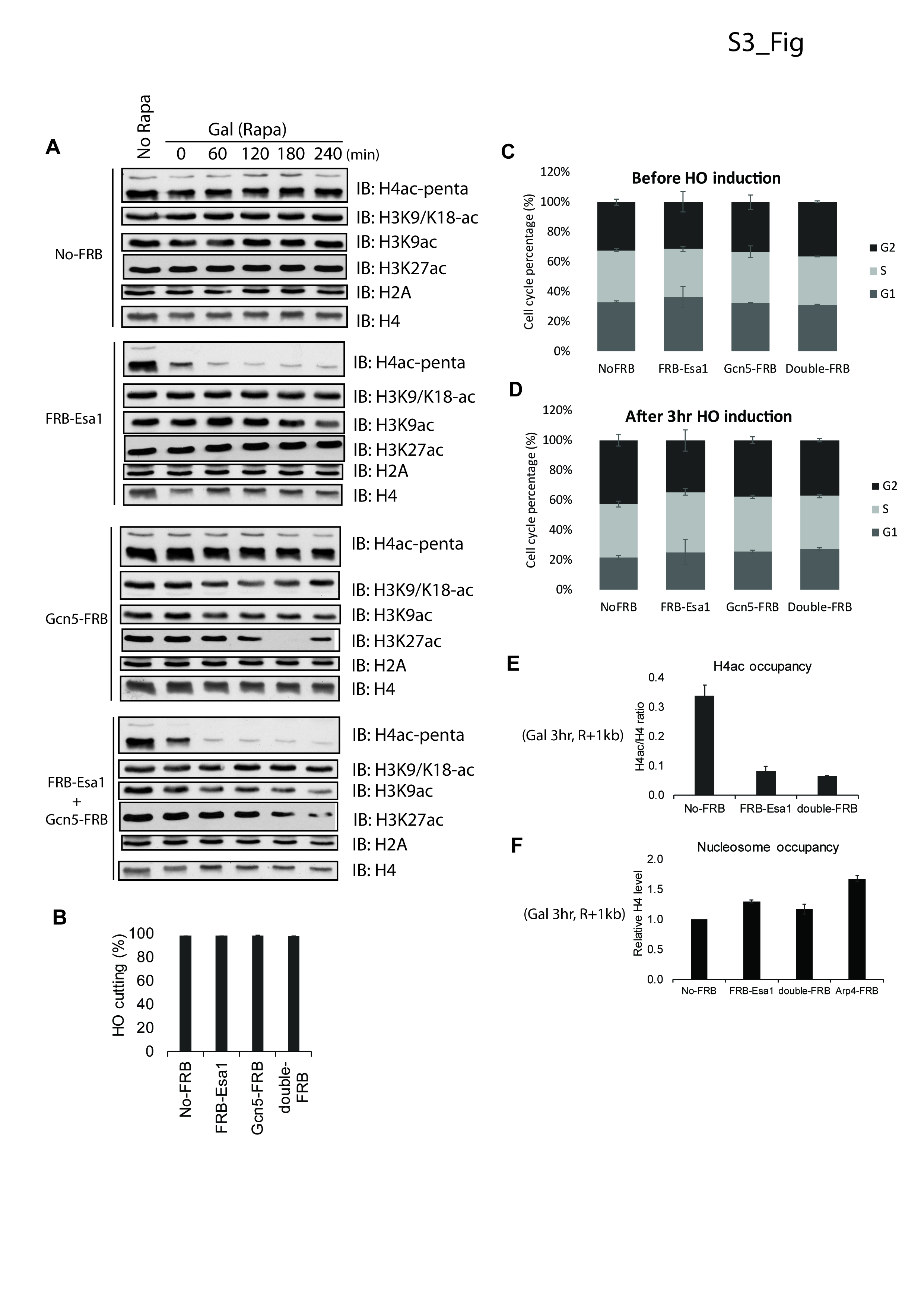

Supplement: S3 Fig — Related to Fig 4. A) Western blot analysis of bulk histone acetylation marks in No-FRB, FRB-Esa1, Gcn5-FRB and FRB-Esa1/Gcn5-FRB cells after 0, 60, 120, 180 and 240min in Galactose to induce the HO break as well as in the presence of rapamycin (added 20min before time 0 of galactose). A no rapamycin control before galactose is also shown. Esa1 depletion shows rapid loss of H4ac (penta) while Gcn5 depletion shows a decrease of H3ac, most notably H3K27ac. B) Percentage of HO cutting efficiency in No-FRB, FRB-Esa1, Gcn5-FRB and FRB-Esa1/Gcn5-FRB strains after 30min of induction in galactose. Error bars represent standard error from biological triplicates. Error bars represent standard errors from biological triplicates. C-D) Cell cycle analysis of cells from the same indicated strains by cell cytometry after fixing and staining with PI, before (C) and after 3hr induction of HO (D) in presence of rapamycin, showing no major changes in cell cycle profiles between strains and conditions. Error bars represent range from biological duplicates. E) ChIP-qPCR of H4ac signal (ratio of H4ac/total H4%IP/input) next to the HO break in No-FRB, FRB-Esa1 and FRB-Esa1/Gcn5-FRB strains after 3hr of galactose induction of HO in presence of rapamycin, showing the expected loss of H4ac. F) ChIP-qPCR of relative H4 level (nucleosome occupancy) in the indicated strains after 3hr galactose induction in presence of rapamycin. Values (% IP/input) near the HO break were compared to the No-FRB samples set to 1, showing an increase in Esa1/Gcn5-depeleted cells. Arp4-FRB is shown as control as it is a shared subunit not only of NuA4 but also INO80/SWR1 chromatin remodeling complexes. Error bars represent range from biological duplicates. (TIF) [file pgen.1009459.s003.tif]

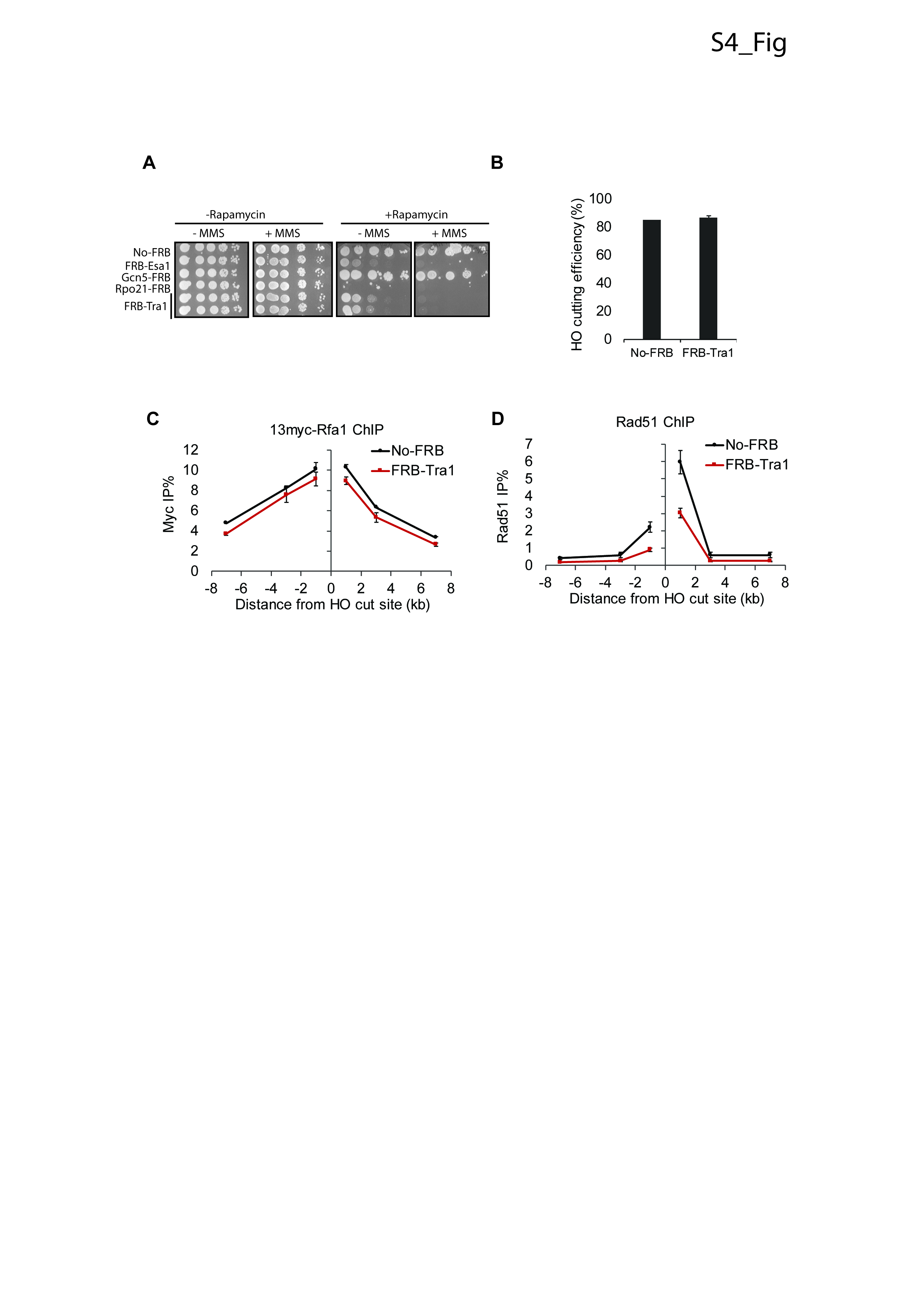

Supplement: S4 Fig — Related to Figs 4 and 5. A) 10-fold serial dilutions of indicated FRB-tagged strains were spotted on solid medium without or with 1μg/ml rapamycin supplemented without or with 0.03% MMS and grown at 30°C. Depletion of the Tra1 subunit shared by NuA4 and SAGA leads to much decreased viability, while not as much as Esa1/Rpo21, but also clear sensitivity to DNA damage. B) Percentage of HO cutting efficiency in No-FRB and FRB-Tra1 strains after 30min of induction in galactose. Error bars represent standard error from biological triplicates. C-D) ChIP-qPCR assay of Rfa1-13myc (C) and Rad51 (D) (% of IP/input at different locations around the HO DSB) in No-FRB and FRB-Tra1 strains after 3hr of galactose induction. Error bars represent range from biological duplicates. (TIF) [file pgen.1009459.s004.tif]

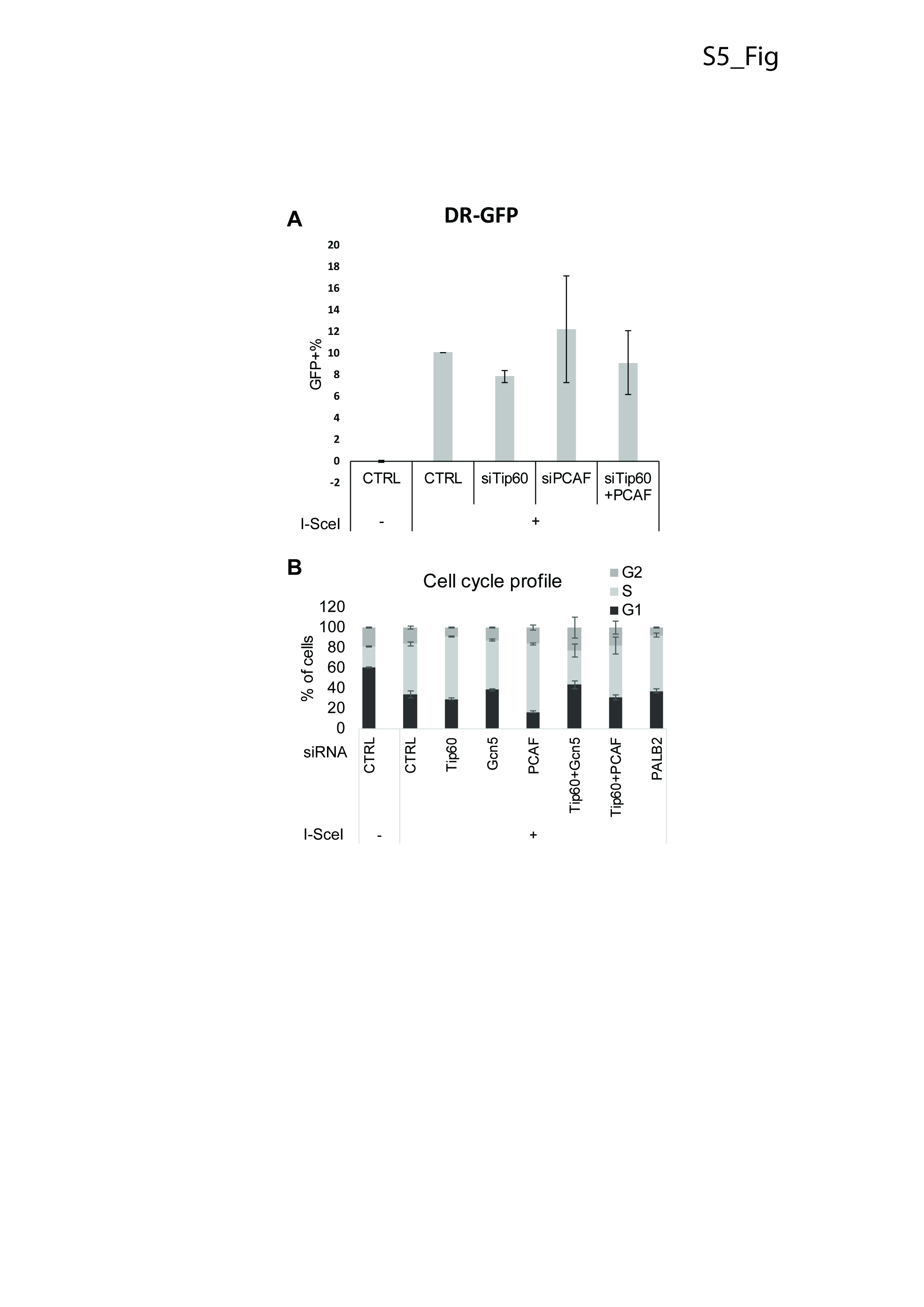

Supplement: S5 Fig — Related to Fig 5D. A) DR-GFP as in Fig 5D with indicated siRNAs. B) Cell cycle analysis of cells shown in (A) and Fig 5D by cell cytometry after fixing and staining cells with PI. Error bars represent range from biological duplicates. Note that siPCAF cells show accumulation in S/G2, potentially accounting for elevated HR repair efficiency observed in these cells. (TIF) [file pgen.1009459.s005.tif]
