# Supplementary material for: NuA4 and SAGA acetyltransferase complexes cooperate for repair of DNA breaks by homologous recombination
Source: PLoS Genet. 2021 Jul 6;17(7):e1009459. doi: 10.1371/journal.pgen.1009459 (PMC8284799; doi:10.1371/journal.pgen.1009459)
Supplement: S1 Table — (DOCX) [file pgen.1009459.s006.docx]

**S1 Table.** **Yeast strains used in this study**.

| **Strain** | **Genotype** | **Reference** |
| --- | --- | --- |
| JKM139 | *MAT*a *hoΔ hmlΔ::ADE1 hmrΔ::ADE1 ade1-100 leu2-3,112 trp1::hisG’ lys5 ura3-52 ade3::GAL::HO* | [[23](#_ENREF_23)] |
| JKM179 | *MAT*α *hoΔ hmlΔ::ADE1 hmrΔ::ADE1 ade1-100 leu2-3,112 trp1::hisG’ lys5 ura3-52 ade3::GAL::HO* | [[23](#_ENREF_23)] |
| QY3501 | JKM139 *esa1-L254P*::NatMX | this study |
| QY3530 | JKM139 *fpr1Δ*::HphMX *tor1-1::URA3* *RPL13A-2*FKBP12::TRP1* (No-FRB) | this study |
| QY3572 | Isogenic to QY3530 except FRB*-ESA1*::KanMX | this study |
| QY3534 | Isogenic to QY3530 except *ESA1*-FRB::KanMX | this study |
| QY3562 | Isogenic to QY3530 except *RPO21*-FRB::KanMX | this study |
| QY3531 | Isogenic to QY3530 except *YNG2*-FRB::KanMX | this study |
| QY3535 | Isogenic to QY3530 except *EAF1*-FRB::KanMX | this study |
| LSY-2900-13A | Symington donor strain wt | [[58](#_ENREF_58)] |
| GA2321 | JKM179 *MATa-inc-URA3* | [[33](#_ENREF_33)] |
| GA2368 | JKM179 *MATa-inc-URA3* *rad52::LEU2* | [[33](#_ENREF_33)] |
| QY3520 | JKM179 *MATa-inc-URA3 esa1-L254P*::NatMX | this study |
| QY3668 | JKM139 *fpr1Δ::HphMX tor1-1 RPL13A-2**FKBP12*::TRP1 MATα-inc-HIS3::URA3* | this study |
| QY3672 | Isogenic to QY3668 except *EAF1*-FRB::KanMX | this study |
| QY3669 | Isogenic to QY3668 except FRB*-Esa1*::KanMX | this study |
| QY3670 | Isogenic to QY3668 except *GCN5*-FRB::KanMX | this study |
| QY3671 | Isogenic to QY3668 except FRB*-Esa1*::KanMX *GCN5*-FRB::NatMX | this study |
| QY3686 | JKM139 *SPT7-*13Myc::*TRP1* | this study |
| QY3692 | JKM139 *SPT7-*13Myc::*TRP1* *xrs2Δ*::KanMX | this study |
| QY3608 | JKM139 *fpr1Δ*::HphMX *tor1-1::URA3* *RPL13A-2*FKBP12::TRP1 Leu2::*BrdU-inc | this study |
| QY3611 | Isogenic to QY3608 except *FRB-Esa1*::KanMX | this study |
| QY3615 | Isogenic to QY3608 except *Gcn5-FRB*::KanMX | this study |
| QY3616 | Isogenic to QY3608 except *FRB-Esa1*::KanMX *Gcn5-FRB*::NatMX | this study |
| QY3596 | Isogenic to QY3530 except FRB*-Tra1*::KanMX | this study |
| HHY168 | K14708 *RPL13A*-2×FKBP12::*TRP1* | [[24](#_ENREF_24)] |
| QY3599 | K14708 *RPL13A*-2×FKBP12::*TRP1 leu2Δ::*HphMX | this study |
| QY3621 | Isogenic to QY3559 except *EAF1-FRB*::KanMX | this study |
| QY3607 | Isogenic to QY3559 except *DNL4-FRB*::KanMX | this study |
| QY3622 | Isogenic to HHY168 except *EAF1-FRB*::KanMX | this study |
| QY3625 | Isogenic to HHY168 except *sae2Δ::HIS3* | this study |
| QY3633 | Isogenic to HHY168 except *exo1Δ::*HphMX | this study |
| QY3624 | Isogenic to HHY168 except *sgs1Δ::HIS3* | this study |
| QY3623 | Isogenic to HHY168 except *fun30Δ::HIS3* | this study |
| QY3636 | Isogenic to HHY168 except *rad52Δ::HIS3* | this study |
| QY3632 | Isogenic to HHY168 except *EAF1-FRB*::KanMX *sae2Δ::HIS3* | this study |
| QY3634 | Isogenic to HHY168 except *EAF1-FRB*::KanMX *exo1Δ::*HphMX | this study |
| QY3631 | Isogenic to HHY168 except *EAF1-FRB*::KanMX *sgs1Δ::HIS3* | this study |
| QY3630 | Isogenic to HHY168 except *EAF1-FRB*::KanMX *fun30Δ::HIS3* | this study |
| QY3637 | Isogenic to HHY168 except *EAF1-FRB*::KanMX *rad52Δ::HIS3* | this study |
| QY3563 | JKM139 +(pRS424 *TRP1 2μ ori)* | this study |
| QY3564 | JKM139 +(pRS424 *EXO1* *TRP1 2μ ori)* | this study |
| QY3565 | JKM139 +(pRS424 *EXO1-D173A* *TRP1 2μ ori)* | this study |
| QY3566 | JKM139 *esa1-L254P*::NatMX +(pRS424 *TRP1 2μ ori)* | this study |
| QY3567 | JKM139 *esa1-L254P*::NatMX +(pRS424 *EXO1* *TRP1 2μ ori)* | this study |
| QY3568 | JKM139 *esa1-L254P*::NatMX +(pRS424 *EXO1-D173A* *TRP1 2μ ori)* | this study |
| QY3569 | JKM139 *fun30Δ*::KanMX +(pRS424 *TRP1 2μ ori)* | this study |
| QY3570 | JKM139 *fun30Δ* +(pRS424 *EXO1* *TRP1 2μ ori)* | this study |
| QY3571 | JKM139 *fun30Δ* +(pRS424 *EXO1-D173A* *TRP1 2μ ori)* | this study |
