# Supplementary material for: NuA4 and SAGA acetyltransferase complexes cooperate for repair of DNA breaks by homologous recombination
Source: PLoS Genet. 2021 Jul 6;17(7):e1009459. doi: 10.1371/journal.pgen.1009459 (PMC8284799; doi:10.1371/journal.pgen.1009459)
Supplement: Attachment — Submitted filename: ResponsetoReviewers.pdf [file pgen.1009459.s008.pdf]

<u>Response to reviewers' comments:</u>
Reviewer #1:
The article by Cheng et al describes new insights to the role of NuA4, in cooperation with SAGA in DSB repair. Interestingly these effects are both at the broken end, regulating resection and Rad51 recruitment, and at the donor sequence where strand invasion occurs. The authors generate and validate some nice tools for analysis of NuA4 function that complements their work with ts-alleles (e.g. FRB-Esa1). Most importantly they define a exciting new cooperative mechanism between SAGA and NuA4 in regulating end resection. The results with the doubly-depleted cells are among the strongest and most compelling in the paper. They also nicely link these changes in acetylation to downstream effects on chromatin remodeler recruitment and to non-histone substrates (RPA). Overall, I really liked this study and think it contributes important new information. Below I highlight a few potential improvements.

We thank the reviewer for the very positive comments, finding our results strong, compelling and exciting.

1. While the trends are clear enough, the authors need to apply some kind of statistical comparison to data in Figure 1 to highlight differences. This is true for all quantitative tests in the paper – some statistical analysis is needed, but I did not see any. Given the subtle effects, there may not be significant differences between the single mutants and WT, but I think the strength of the results in Figure 4 and 5 still make the most important contribution.

As suggested by the reviewer and the editors, we have now applied statistical analysis to the quantitative tests in the paper (T-test). Differences compared to wild type/control conditions that are discussed in the text are now supported by statistical analysis showing that they are significant.

2. I did not follow the logic on line 132 that decreased Rad51 recruitment supports a role for NuA4 after resection. Wouldn't reduced resection lead to shorter RPA filaments and therefore less Rad51 signal in bulk ChIP experiments? I think this could be reworded.

We agree that decreased Rad51 recruitment measured by ChIP can be a consequence of reduced end resection/shorter RPA filament. This sentence has been modified to reflect that.

3. There are no molecular weight markers on any of the western blots in the paper. In Figure 5C it is not even clear which RPA subunit is being blotted? Which protein is modified? In addition, can the authors say more about acetylation of RPA? I wasn't aware of this modification and I wondered if it cooperates with RPA sumoylation or phosphorylation that can be modified by DNA damage.

We are sorry about this and have now included MW marker positions. RPA acetylation was described and analyzed in our previous paper (Cheng et al. PNAS 2018). We did not investigate a relationship with phosphorylation or sumoylation but showed that RPA1 acetylation by NuA4 was affecting binding to ssDNA. We have now included additional information on this.

4. Histone acetylation promotes transcription in other contexts. Transcription has recently been

ascribed various important roles in DSB repair. For example, RNA:DNA hybrids are implicated in end resection, RNAPIII and the MRN/X complex have both been implicated in regulating transcription at DSB ends. The authors should say more about these connections to their work and consider whether the acetylation effects are important for driving transcription near breaks. See PMIDs: 33406426; 33626331 and other slightly older work on transcription-associated HR (TA-HRR).

We thank the reviewer for this suggestion. It could be indeed interesting to investigate this connection since NuA4 and SAGA are clear co-activators of transcription. We added this possible connection in the discussion.

Reviewer #2:
Cheng, Cote and Cote investigate the roles of two KAT complexes, NuA4 and SAGA, in DSB in yeast. NuA4 has been demonstrated to be recruited to DSBs and to aid in recruitment of both repair complexes and chromatin remodeling complexes. Here the authors aim to further delineate these functions using a conditional mutation and a rapid depletion approach. They also compare the effects of Esa1 (NuA4) and Gcn5 (SAGA) alone and together. Although these approaches are useful and may give new insights, several issues limit the impact of the paper as currently presented.

We understand that the reviewer saw the potential of our study and approaches, but presenting more background/technical information would help the perceived impact of the paper to a larger non-specialist audience.

1. Although many of assays (e.g. HO-induced DSBs) are well-established, they still should be described well enough that newcomers can understand what the assay is and why it is used. The descriptions of all assays in Figure 1 need to be more clearly explained in the text, to set the stage for that figure and subsequent figures in the paper.

We made an effort to provide more background information and explanation about the assays presented in Figure 1.

2. Use of the rapid degrader system is a good approach. However, I could not find a blot showing the timing and level of knock down for FRB-Esa1 or FRB-Gcn5. These should be added to the blots shown in Suppl 3B. This is especially important for Gcn5, since there seems to be little or no effect on H3Ac in the FRB-Gcn5 strain.

As explained during our email discussion with the editors, the anchor-away system is not a rapid degrader system. It is designed to deplete protein from the nucleus by forcing their translocation to the cytoplasm. This is now explained in the text. The validation of the system for specific nuclear proteins is done, after confirmation of genetic alteration, by phenotypic analysis in condition of depletion corresponding to known effect of traditional knock-out or mutations. Here, slow growth, sensitivity to DNA damage and effect on chromatin acetylation are used. Specifically for Gcn5-FRB, it is known that its acetylation targets on H3 often overlap with those of the Sas3 acetyltransferase, needing to get rid of both to lose bulk acetylation (Howe et al.

G&D 2001). We added a new target in our WB analysis of bulk acetylation in conditions of depletion. H3K27 acetylation is mostly dependent on Gcn5 and we see a clear loss of this mark in our Gcn5-FRB background after depletion from the nucleus (updated Figure S4A).

3. Th effects of the esa1 ts mutant on resection are described (line 116) as "small but significant". How was significance established? Also, in Fig 1E, the red line (mutant) is higher than the black line (WT)—doesn't that indicate more, not less, resection in the mutant?

In Fig. 1E, the amount of DNA is measured at different locations near the break. The fact that the mutant has more DNA left near the break (red line) compared to WT conditions (black line) indicates that there is less resection occurring after double-strand break formation. The text was modified to explain this more clearly.

4. The "significance" question is relevant to all assays and time points, in all graphs, in Fig 1.

As indicated above for reviewer 1 first comment, we have now applied statistical comparison to the quantitative tests in the paper (T-test). Differences compared to wild type/control conditions that are discussed in the text are now supported by statistical analysis showing that they are significant.

5. A brief introduction to the resection factors used in the genetic assays in Fig 2 would also help the non-expert reader. Just a line or two about what the factors are what steps they are known to execute in resection.

We thank the reviewer for the suggestion, and we have now added the requested information.

6. The effects of the double esa1 gcn5 mutants are interesting, but these KATs affect many processes, including transcription and cell cycle progression, in addition to DNA repair. The authors need to demonstrate that the effects are not secondary to the cells being really "sick"—i.e. show some process that is NOT affected by the double mutant. Or show that the effects are not coupled to or downstream of transcriptional defects. They should also directly address exactly how sick the double mutants are.

We agree with the reviewer that double *esa1 gcn5* mutants affect many processes. In fact, it has been known for a long time that they are synthetic lethal/non-viable, just like a combination of histone H3 and H4 acetylation mutants. That is exactly the reason why the anchor-away system is so useful here, as we can deplete both proteins very rapidly from the nucleus compartment before secondary cellular effects appear. We have already included as control the level of DSB formation by the HO endonuclease which is a readout of possible transcription defect as the HO gene is under the control of the *GAL* promoter. This control shows that, in our experimental conditions/timeframe, *GAL*-dependent transcription is not affected. In addition, the time-frame/growth conditions used do not allow a significant effect of the double-depletion on the cell cycle (doubling time in raffinose/galactose media is significantly longer than in glucose media, i.e. more than 180min).

7. Minor point—the authors in the beginning point out that NuA4 has been implicated in multiple types of DNA repair. They should also mention other types of repair that involve Gcn5, for example NER in mammalian cells, together with E2F.

As requested, we added the info/reference.

Reviewer #3:
The authors use a conditional mutant and rapid depletion approach along with an integrated double strand-break induction system to show, in yeast, that SAGA and NuA4 cooperate to acetylate chromatin around sites of DNA damage and at donor sites. Cooperation between these chromatin modifying complexes is important for DNA end resection to occur, recruitment of chromatin remodeling complexes, acetylation of DNA repair factors, and homologous recombination.
This work was a pleasure to read and the discussion clearly points where the next papers should go. Comments on minor issues are as follows:

Of course, we thank the reviewer for his very positive comments.

Minor points:
1. Make the Y-axis for Figures 1 A and B the same, so they can be easily compared.

This has now been done (see modified Fig1 A-B).

2. "Interestingly, both systems indicate that the domain of NuA4-dependent increased H4 acetylation upon DSB formation is larger than the extent of NuA4 spreading in these conditions (3-5kb, see [5]), suggesting longer-range contact in the tri-dimensional space."
a. Could the authors explain this more? How would there be acetylation without NuA4 spreading? There is a distinction being made between spreading and contact, which implies NuA4 is responsible for acetylation in regions where it can not be shown to interact.

This is of course an interesting point that raises questions about 3D organisation of the genome around the DNA break. There are other examples in the literature about chromatin factors having long-range effect away from their detected binding sites detected by ChIP. This has been added to the text.

3. "Accordingly, in the ts mutant, we detect a small but significant decrease of DNA end resection near the break site as well as decreased single-strand DNA (ssDNA) binding RPA and Rad51 recombinase recruitment (Fig. 1E-G)."
a. Figure 1E shows a small increase in DNA end resection near the break site. Are the labels wrong? The data in 1H supports the text and contradicts 1E.
b. Figure 1F is labeled Myc-Rfa1, and so does the figure legend, but the manuscript text refers to RPA

a. We apologize about the confusion about Fig. 1E. As stated above for Reviewer 2, the amount of DNA is measured at different locations near the break. The fact that the mutant has more DNA left near the break (red line) compared to WT conditions (black line) indicates that there is less resection occurring after double-strand break formation. The text was modified to explain this more clearly.
b. Rfa1 is the largest subunit of the ssDNA-binding trimeric RPA complex. So in our ChIP experiments it is used to reflect RPA binding. This has now been more clearly indicated in the text.

4. It is interesting that the Rfa data in Figure 1 shows decreased ssDNA near and far from the break point, but the Rad51 data shows it more near the break site. Please comment on this.

Rad51 filament loading on resected ssDNA occurs by initial nucleation near the break through replacement of RPA. This explains the different pattern of binding between RPA and Rad51 detected by ChIP-qPCR (Rad51 being enriched closer to the break).

5. "(Fig. 3F). These results provide the first evidence that links NuA4 to D-loop formation" The term "links" suggests some need or influence of NuA4, but at this point in the paper, you have shown that NuA4 and H4ac are present at the site of D-loop formation.

This is a good point of the reviewer and we changed the text as "…physically links NuA4 to the formation of the D-loop, suggesting a role…".

6. Quantify 4E and 5C

We understand the request, but these data were obtained by classical ECL on membrane after gel transfer followed by exposure on films. Thus, with the well-known non-linearity of ECL signals, we feel that strict quantification of bands on films will not reflect real numerical fold-changes on proteins *in vivo*. We prefer to leave these as qualitative data showing clear decreases of phospho-Rad53 and acetyl-Rfa1 signals.
