# Supplementary material for: NuA4 and SAGA acetyltransferase complexes cooperate for repair of DNA breaks by homologous recombination
Source: PLoS Genet. 2021 Jul 6;17(7):e1009459. doi: 10.1371/journal.pgen.1009459 (PMC8284799; doi:10.1371/journal.pgen.1009459)
Supplement: Attachment — Submitted filename: 2ndrevisedNuA4-SAGA-HRcoverletterPLoS.pdf [file pgen.1009459.s009.pdf]

AXE ONCOLOGIE

CENTRE DE RECHERCHE CHU de Québec Université Laval

Directeur
Dr Jacques Côté

Directeur Adjoint
Dr Martin Simard

Chercheurs
Dr Alan Anderson
Dr Louis Archambault
Dr Dimcho Bachvarov
Dre Isabelle Bairati
Dr Luc Beaulieu
Dr Jean-Mathieu Beauregard
Dr Steve Bilodeau
Dr Nicolas Bisson
Dr François Bordeleau
Dr Jacques Brisson
Dr Manuel Caruso
Dr Jean Charron
Dre Jocelyne Chiquette
Dre Anne Dagnault
Dre Christine Desbiens
Dr Jean-François Desbiens
Dre Michèle Desjardins
Dr Philippe Després
Dre Caroline Diorio
Dr Michel Dorval
Dr Serge Dumont
Dre Lise Fillion
Dr Sébastien Fortin
Dr Vincent Fradet
Dr Yves Fradet
Dre Amélie Fradet-Turcotte
Dr Bruno Gagnon
Dr Pierre Gagnon
Dr René C. Gaudreault
Dre Lynn Gauthier
Dr Marc-Étienne Huot
Dr Samer Hussein
Dre Lucie Jeannotte
Dr Louis Lacombe
Dr Patrick Laprise
Dre Mélanie Laurin
Dre Isabelle Laverdière
Dre Josée N. Lavoie
Dre Mireille Lavoie
Dre Julie Lemieux
Dr Jean-Yves Masson
Dre Elizabeth Maunsell
Dr Rachid Mazroui
Dr Thomas Moss
Dr Hermann Nabi
Dr Amine Nourani
Dre Marie Plante
Dre Brigitte Poirier
Dr Éric Poirier
Dr Guy Poirier
Dr Frédéric Pouliot
Dre Louise Provencher
Dre Josée Savard
Dr Paul Toren
Dr Éric Vigneault

Quebec City, June 16th 2021

Editors, PLoS Genetics

Dear Editors,

Please find enclosed our further revised manuscript entitled " **NuA4 and SAGA acetyltransferase complexes cooperate for repair of DNA breaks by homologous recombination**" by Cheng *et al.*, for publication in *PLoS Genetics*.

We are of course delighted that all 3 reviewers recommended acceptance, found our revised manuscript clearer and much improved, as well accessible and of interest for a wide audience. Previous concerns and suggestions were addressed, and statistics were added.

As suggested by reviewer 1, we have now additionally performed one-way ANOVA analysis followed by post-hoc test (Tukey) for the data presented in Figure 3 where two bars are compared to the first one. This was also done for the other similar situations in Figures 4C-D and 5B and D. In all cases, the increases or decreases of signals compared to control conditions remain statistically significant as initially determined by one-tail T-test. This is now indicated in the figure legends.

We also followed reviewer 1's second suggestion and changed the wording of the text related to Figure 3F reflecting that the detected increase of acetylation is not statistically significant.

We are looking forward to your final acceptance of this study in *PLoS Genetics*. Thank you for your time on this submission.

Sincerely,

Jacques Côté, Ph.D.
Professor of Molecular Biology, Laval University
Canada Research Chair in Chromatin Biology and Molecular Epigenetics
jacques.cote@crhdq.ulaval.ca; http://www.crc.ulaval.ca/jacques_cote

Ça se découvre ici.

Axe Oncologie, Centre de recherche du CHU de Québec-Université Laval
9, rue McMahon, Québec, Canada, G1R 3S3 • 418 525-4444 #15281
