# Supplementary material for: NuA4 and SAGA acetyltransferase complexes cooperate for repair of DNA breaks by homologous recombination
Source: PLoS Genet. 2021 Jul 6;17(7):e1009459. doi: 10.1371/journal.pgen.1009459 (PMC8284799; doi:10.1371/journal.pgen.1009459)
Supplement: Attachment — Submitted filename: cote plos genetics.docx [file pgen.1009459.s007.docx]

The authors use a conditional mutant and rapid depletion approach along with an integrated double strand-break induction system to show, in yeast, that SAGA and NuA4 cooperate to acetylate chromatin around sites of DNA damage and at donor sites. Cooperation between these chromatin modifying complexes is important for DNA end resection to occur, recruitment of chromatin remodeling complexes, acetylation of DNA repair factors, and homologous recombination.

This work was a pleasure to read and the discussion clearly points where the next papers should go. Comments on minor issues are as follows:

Minor points:

1. Make the Y-axis for Figures 1 A and B the same, so they can be easily compared.
2. “Interestingly, both systems indicate that the domain of NuA4-dependent increased H4 acetylation upon DSB formation is larger than the extent of NuA4 spreading in these conditions (3-5kb, see [5]), suggesting longer-range contact in the tri-dimensional space.”
   a. Could the authors explain this more? How would there be acetylation without NuA4 spreading? There is a distinction being made between spreading and contact, which implies NuA4 is responsible for acetylation in regions where it can not be shown to interact.
3. “Accordingly, in the ts mutant, we detect a small but significant decrease of DNA end resection near the break site as well as decreased single-strand DNA (ssDNA) binding RPA and Rad51 recombinase recruitment (Fig. 1E-G).”
   a. Figure 1E shows a small increase in DNA end resection near the break site. Are the labels wrong? The data in 1H supports the text and contradicts 1E.
   b. Figure 1F is labeled Myc-Rfa1, and so does the figure legend, but the manuscript text refers to RPA
4. It is interesting that the Rfa data in Figure 1 shows decreased ssDNA near and far from the break point, but the Rad51 data shows it more near the break site. Please comment on this.
5. “(Fig. 3F). These results provide the first evidence that links NuA4 to D-loop formation” The term “links” suggests some need or influence of NuA4, but at this point in the paper, you have shown that NuA4 and H4ac are present at the site of D-loop formation.
6. Quantify 4E and 5C
